# Author Correction: In vivo clonal expansion and phenotypes of hypocretin-specific CD4^+^ T cells in narcolepsy patients and controls

**DOI:** 10.1038/s41467-019-13776-0

**Published:** 2020-01-08

**Authors:** Wei Jiang, James R. Birtley, Shu-Chen Hung, Weiqi Wang, Shin-Heng Chiou, Claudia Macaubas, Birgitte Kornum, Lu Tian, Huang Huang, Lital Adler, Grant Weaver, Liying Lu, Alexandra Ilstad-Minnihan, Sriram Somasundaram, Sashi Ayyangar, Mark M. Davis, Lawrence J. Stern, Elizabeth D. Mellins

**Affiliations:** 1 Department of Pediatrics–Human Gene Therapy, Stanford University School of medicine, Stanford, CA 94305 USA; 2 Stanford Immunology, Stanford University School of Medicine, Stanford, CA 94305 USA; 3 Department of Pathology, University of Massachusetts Medical School, Worcester, MA 01655 USA; 4 Human Immune Monitoring Center, Stanford University School of Medicine, Stanford, CA 94305 USA; 5 Institute for Immunity, Transplantation and Infection, Department of Microbiology and Immunology, Stanford University School of Medicine, Stanford, CA 94305 USA; 6 Danish Center for Sleep Medicine, Department of Clinical Neurophysiology, Rigshospitalet, 2600 Glostrup, Denmark; 7 Department of Biomedical Data Science, Stanford University School of Medicine, Stanford, CA 94305 USA; 8 Howard Hughes Medical Institute, Stanford University, Stanford, CA 94305 USA; 9 Present Address: UCB Pharma, Slough, SL13WE UK; 10 Present Address: Department of Biological Regulation, Weizmann Institute of Science, Rehovot, 7610001 Israel

**Keywords:** Peripheral tolerance, T-cell receptor, Neuroimmunology, Autoimmune diseases, Sleep disorders

Correction to: *Nature Communications* 10.1038/s41467-019-13234-x, published online 20 November 2019.

The original version of this Article contained an error in the Data Availability statement. The accession code for the X-ray structural data of DQ6-HCRT_56-69_ incorrectly stated ‘PDB ID: 6GIG ‘. The correct version reads ‘PDB ID: 6DIG’.

This has now been corrected in both the PDF and HTML versions of the Article.

