# Supplementary material for: In vivo clonal expansion and phenotypes of hypocretin-specific CD4+ T cells in narcolepsy patients and controls
Source: Nat Commun. 2019 Nov 20;10:5247. doi: 10.1038/s41467-019-13234-x (PMC6868281; doi:10.1038/s41467-019-13234-x)
Supplement: Supplementary file 1 — Supplementary Information [file 41467_2019_13234_MOESM1_ESM.pdf]

Supplementary Information

In vivo clonal expansion and phenotypes of hypocretin-specific $CD4^+$ T cells in narcolepsy patients and controls

Jiang et al.

This document includes 9 supplementary figures and 1 supplementary table.

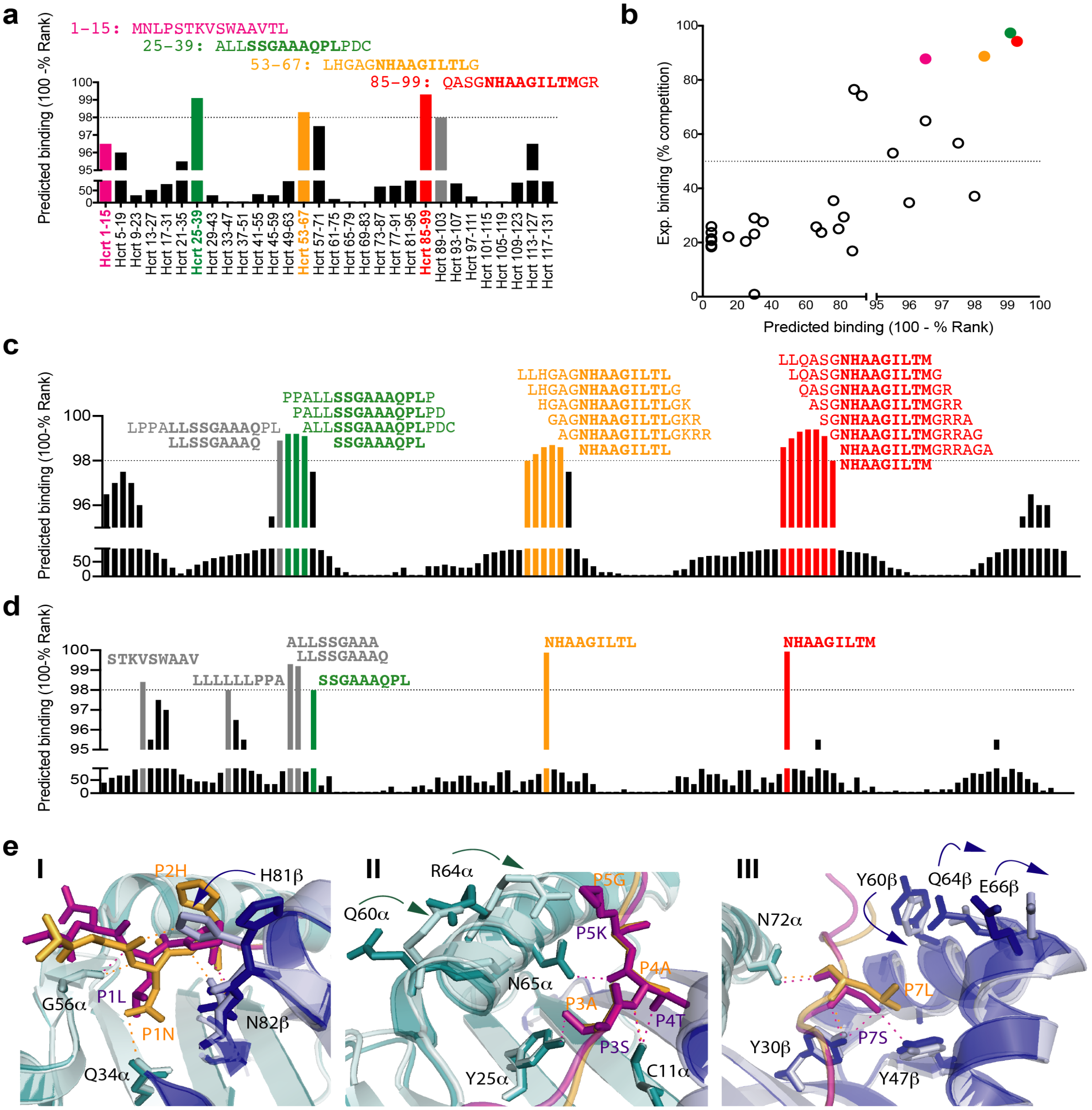

Supplementary Fig. 1 NetMHCIIpan3.2 prediction and structural analysis of HCRT binding to DQ6. (**a**) Predicted binding of the 30 overlapping 15-mer peptides used in the peptide loading assay (Fig. 2a). The %Rank reflects how the predicted affinity for a given peptide ranks compared to a set of 200,000 random natural peptides of the same length[1]. The value (100-%Rank) is positively correlated to the predicted binding affinity. Strong binders ranking in the top 2% are highlighted using a similar color scheme as in Fig. 2a. Gray indicates a predicted strong binder that was not determined by the empirical experiment. $HCRT_{1\text{-}15}$ (in magenta) was predicted as a weak binder. (**b**) A comparison between prediction and the empirical result. Prediction showed 100% sensitivity and specificity when 85% was used as a prediction cutoff and 75% competition was used as the empirical cutoff for DQ6 binding. (**c** and **d**) Predicted DQ6 binding to all possible (c) 15-mer or (d) 9-mer peptides derived from prepro-HCRT. Strong binders as shown were predicted and 9-aa binding cores were bolded. Colors match the color scheme in (a). Gray indicates predicted strong binding cores that are outside of or overlap with the region with strong binders determined by the empirical experiment. Notably, the software failed to predict the X-ray structure-determined core[2], LPSTKVSWA in $HCRT_{1\text{-}15}$ (in blue). (**e**) Zoom-in of the three regions (I-III) shown in Fig. 2c. I) The polar P1N of $HCRT_{56\text{-}69}$ forms three hydrogen (H)–bonds (dotted lines, not in DQ6-$HCRT_{1\text{-}13}$) causing side chain rotations (arrows) at H81β and N82β. II) P3A and P4A of $HCRT_{56\text{-}69}$ abolished three H-bonds observed in DQ6-$HCRT_{1\text{-}13}$ allowing DQ6α1 helix to move inward with side chain rotations at Q60α and R64α. III) The hydrophilic P7L disabled two H-bonds observed in DQ6-$HCRT_{1\text{-}13}$ forcing DQ6β1 helix to move outward as reflected by side chain rotations at Y60β, Q64β and E66β.

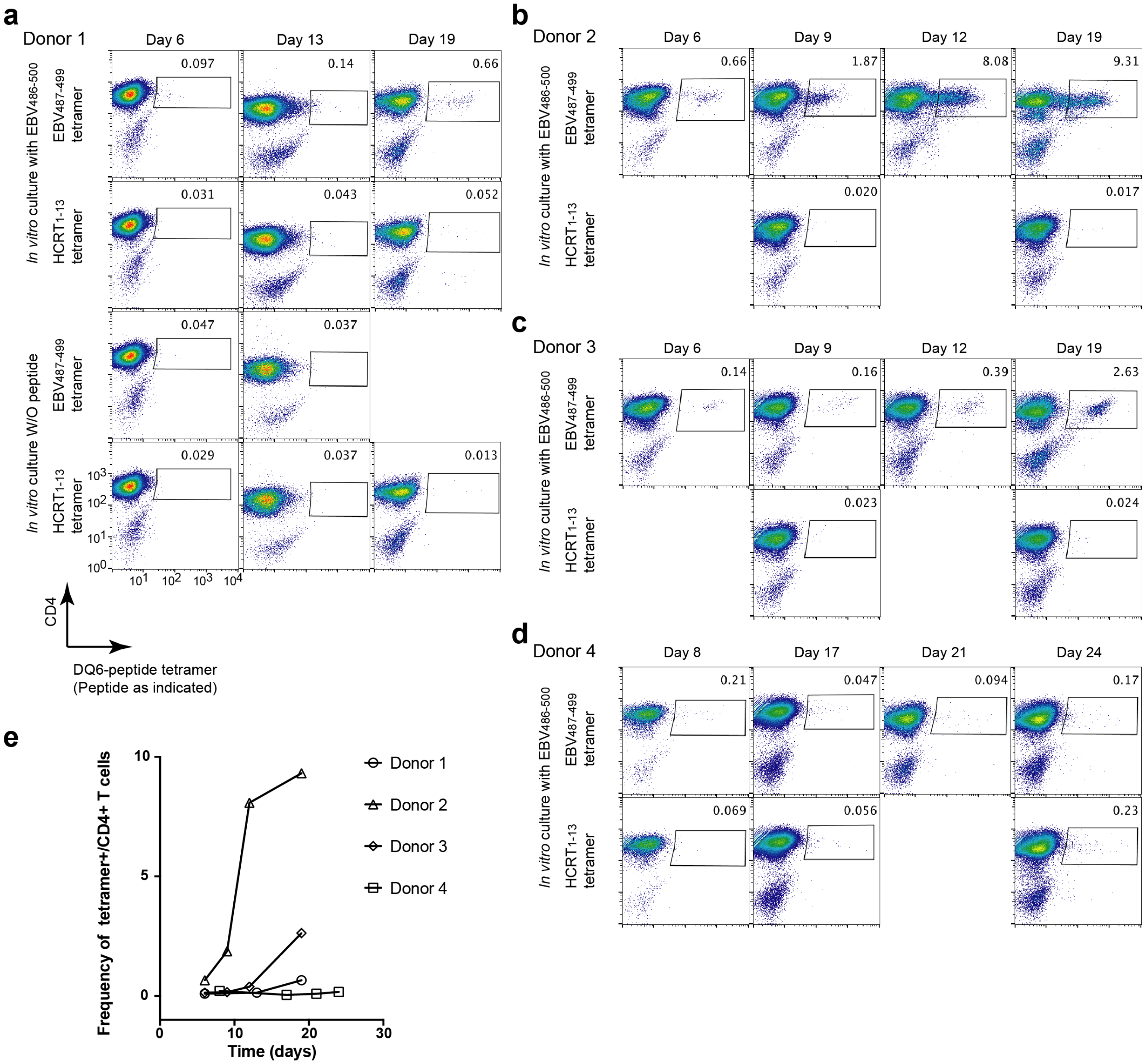

Supplementary Fig. 2 *In vitro* enrichment of DQ6-EBV$_{487\text{-}499}$ tetramer^{+}/CD4^{+} cells. (**a**) Dot-plots showing time dependent enrichment of DQ6-EBV$_{487\text{-}499}$ tetramer^{+}/CD4^{+} T cells after *in vitro* stimulation with the EBV$_{486\text{-}500}$ peptide (see methods). No enrichment was observed for cells binding the control DQ6-HCRT$_{1\text{-}13}$ tetramer or after culture without the EBV peptide. Frequencies (%) of tetramer^{+}/CD4^{+} cells are indicated. (**b-d**) *In vitro* stimulation of cells from other donors suggests that our approach enriches tetramer^{+} T cells from most, but not all donor samples. (**e**) A comparison of time dependent enrichment of DQ6-EBV$_{487\text{-}499}$ tetramer^{+}/CD4^{+} T cells in the four donors. All donors used in the EBV stimulation experiments are narcoleptic patients with unknown EBV infection history.

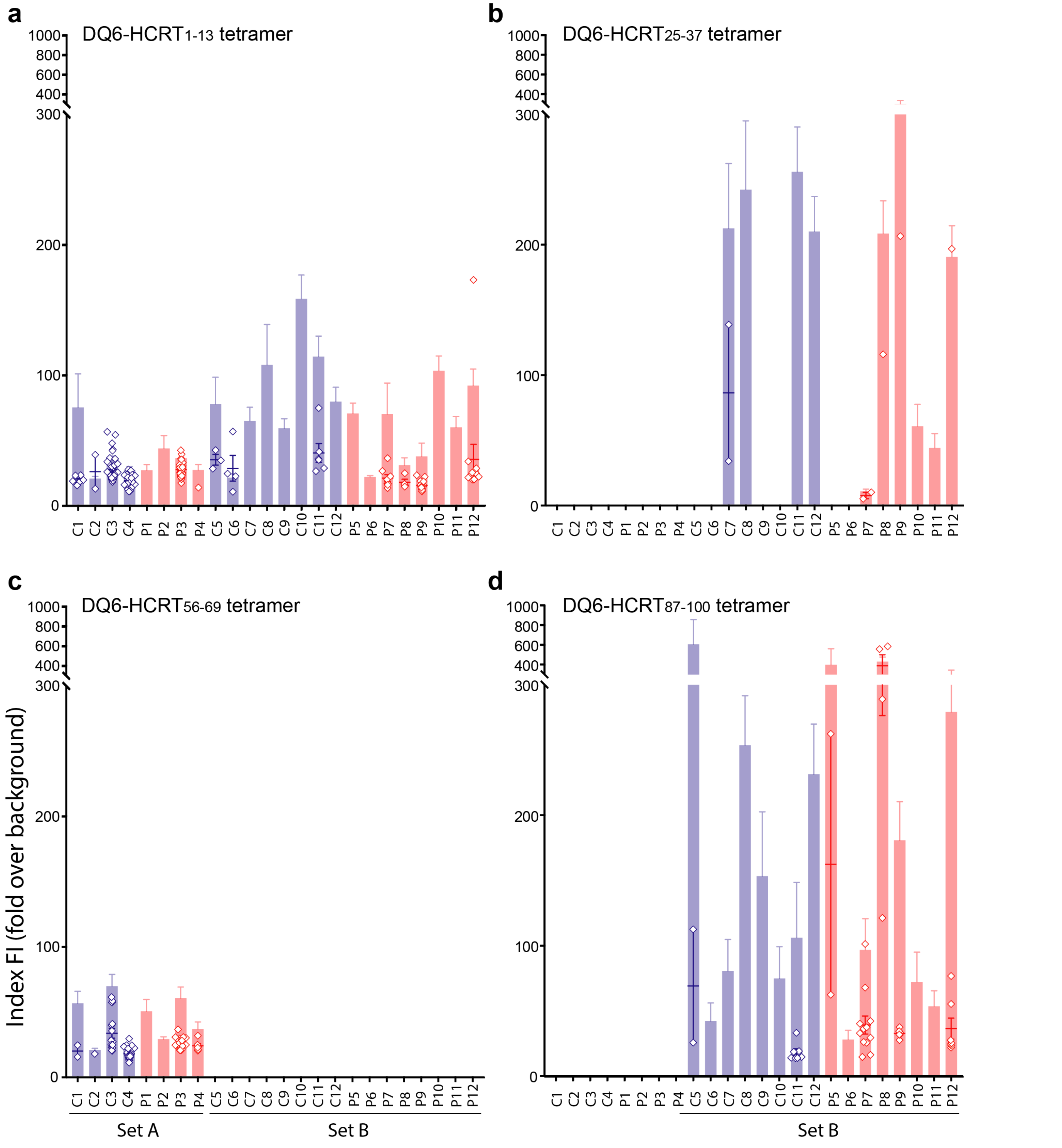

Supplementary Fig. 3 Tetramer binding rank of cells that are expanded *in vivo*. Bar graphs represent the mean ± SEM FI of tetramer-associated signal on the surface of sorted tetramer^{+}/CD4^{+} single cells from the indicated subject-tetramer category (Table 1). Each open symbol represents the index FI of tetramer-associated signal on the surface of one expanded cell identified from the same subject-tetramer category as the corresponding bar graph. Control: blue, patient: red. All values are normalized with background MFI of CD4^{+} population.

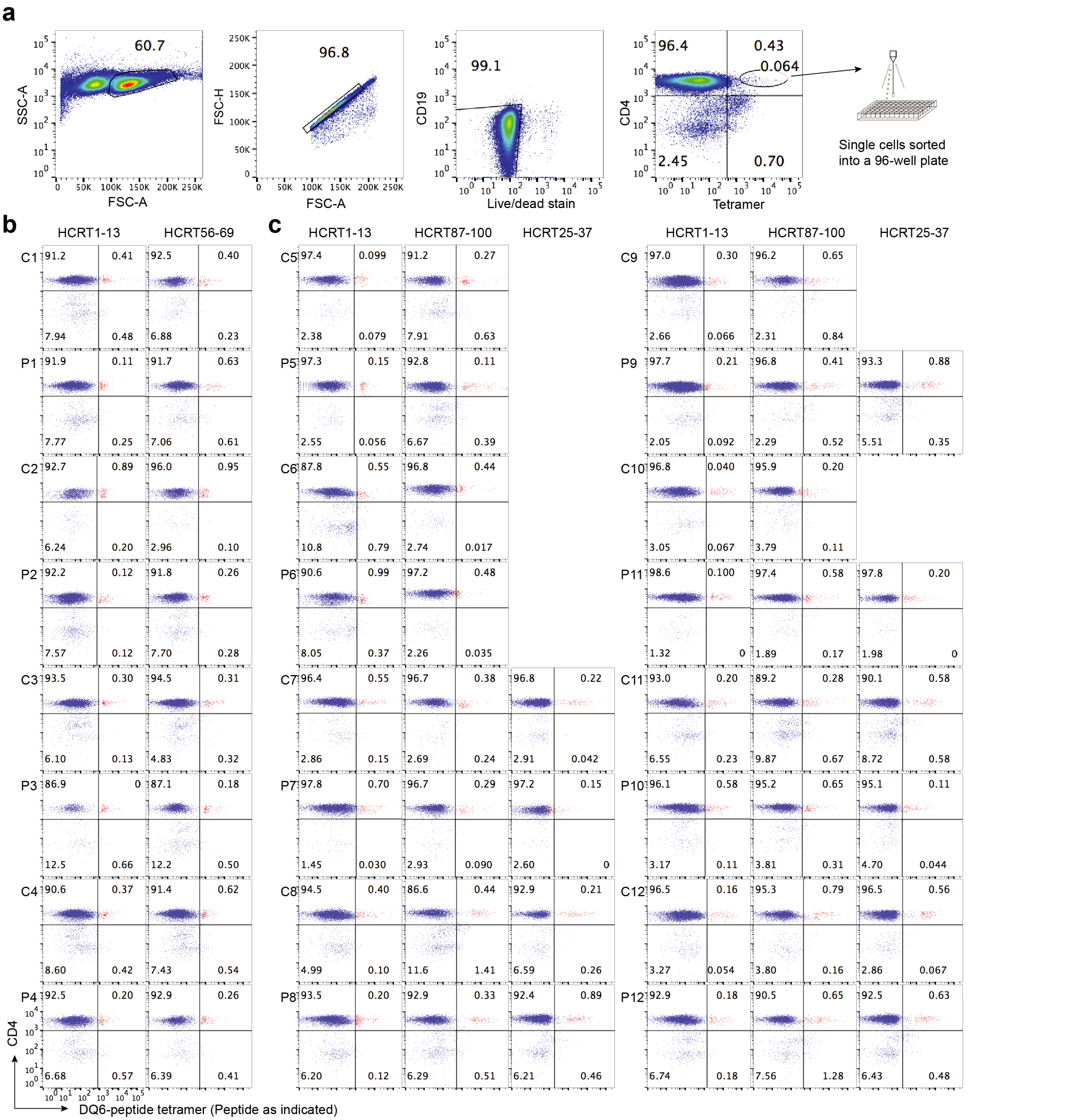

Supplementary Fig. 4 Isolation of DQ6-HCRT$_{peptide}$ tetramer$^+$ cells using iFACS. (**a**) Gating strategy for iFACS. 3-5 million MACS-isolated CD4$^+$ T cells per subject sample were tetramer-stained and screened by iFACS to sort a 96-well plate of single tetramer$^+$ cells. Singlets, live cells, and CD19$^-$/CD4$^+$/tetramer$^+$ cells were gated as indicated. This gating strategy was used for iFACS isolation of single cells that were analyzed in Figure 3e, Table 1, 2, and Supplementary Figure 5. (**b** and **c**) Overlay of sorted single cells (red) and the bulk CD4$^+$ T cells (blue) from each DQ6$^+$ donor (C: control; P: patient). Tetramer specificities are indicated at the top of each column panel.

a

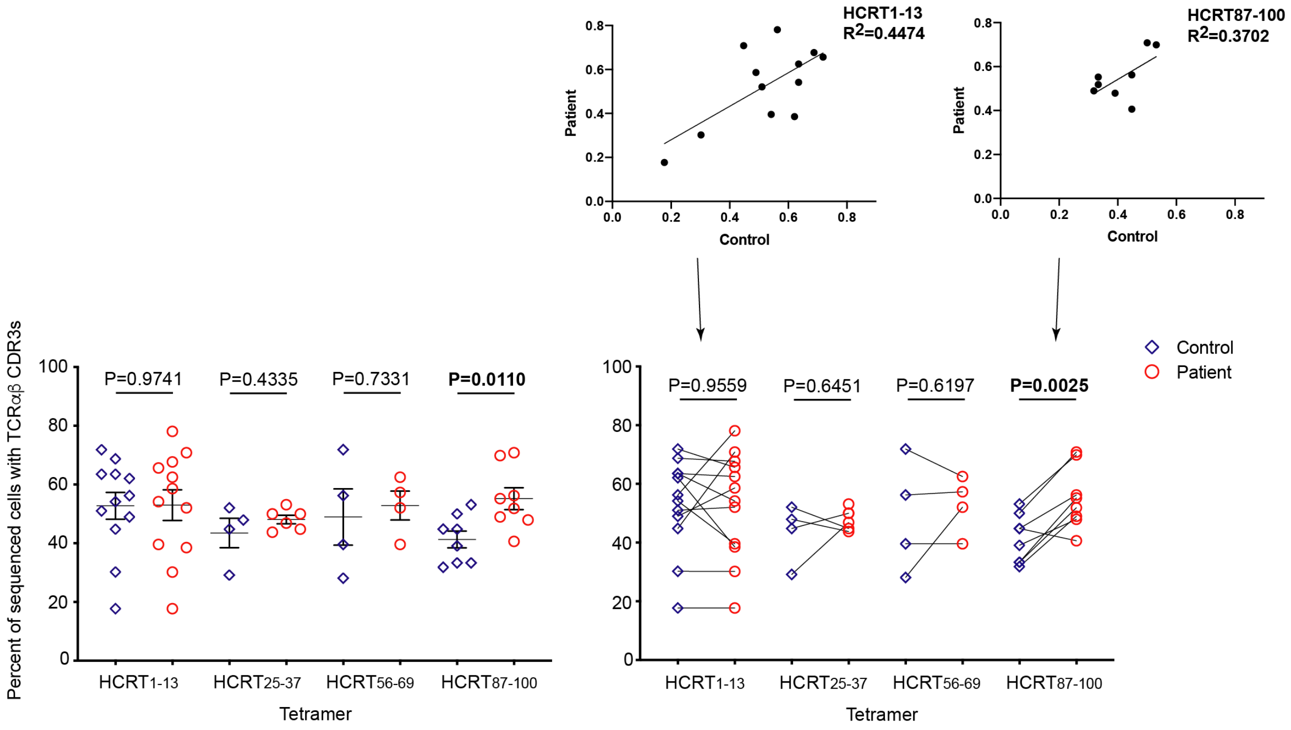

b

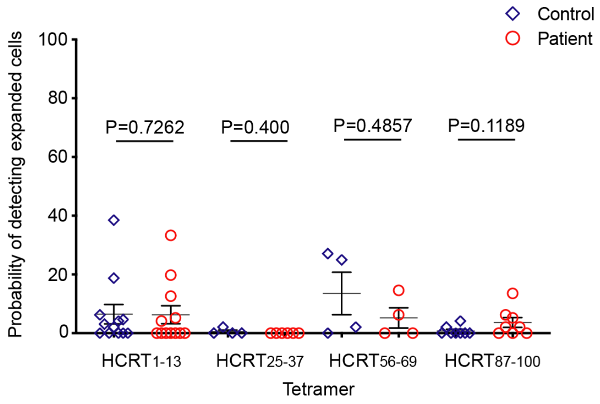

Supplementary Fig. 5 Statistical analysis supporting case/control differences in DQ6-HCRT$_{87\text{-}100}$ tetramer^{+} cells. (**a**) Percent of informative wells that yielded TCRα/β sequences of cells from each subject with the indicated tetramer specificity. Significance (P<0.05, bolded) is determined using an unequal variances *t*-test (left panel) or a paired *t*-test (right panel). The scatter plots (top right panel) show the presence of strong correlations between controls and patients within the pair from the same experiment. The correlation provides the statistical rationale for the paired *t*-test. This analysis shows a biological difference between case and control samples in the DQ6-HCRT$_{87\text{-}100}$ tetramer category. (**b**) Comparison of subjects having expanded tetramer^{+} cells. Significance (P<0.05, bolded) is determined using the Mann-Whitney U test. The error bar represents mean ± SEM. There is a marginal difference (P=0.1189) between patients and controls in the DQ6-HCRT$_{87\text{-}100}$ tetramer category. The skewing of more patients having expanded cells is only observed for the DQ6-HCRT$_{87\text{-}100}$ tetramer specificity.

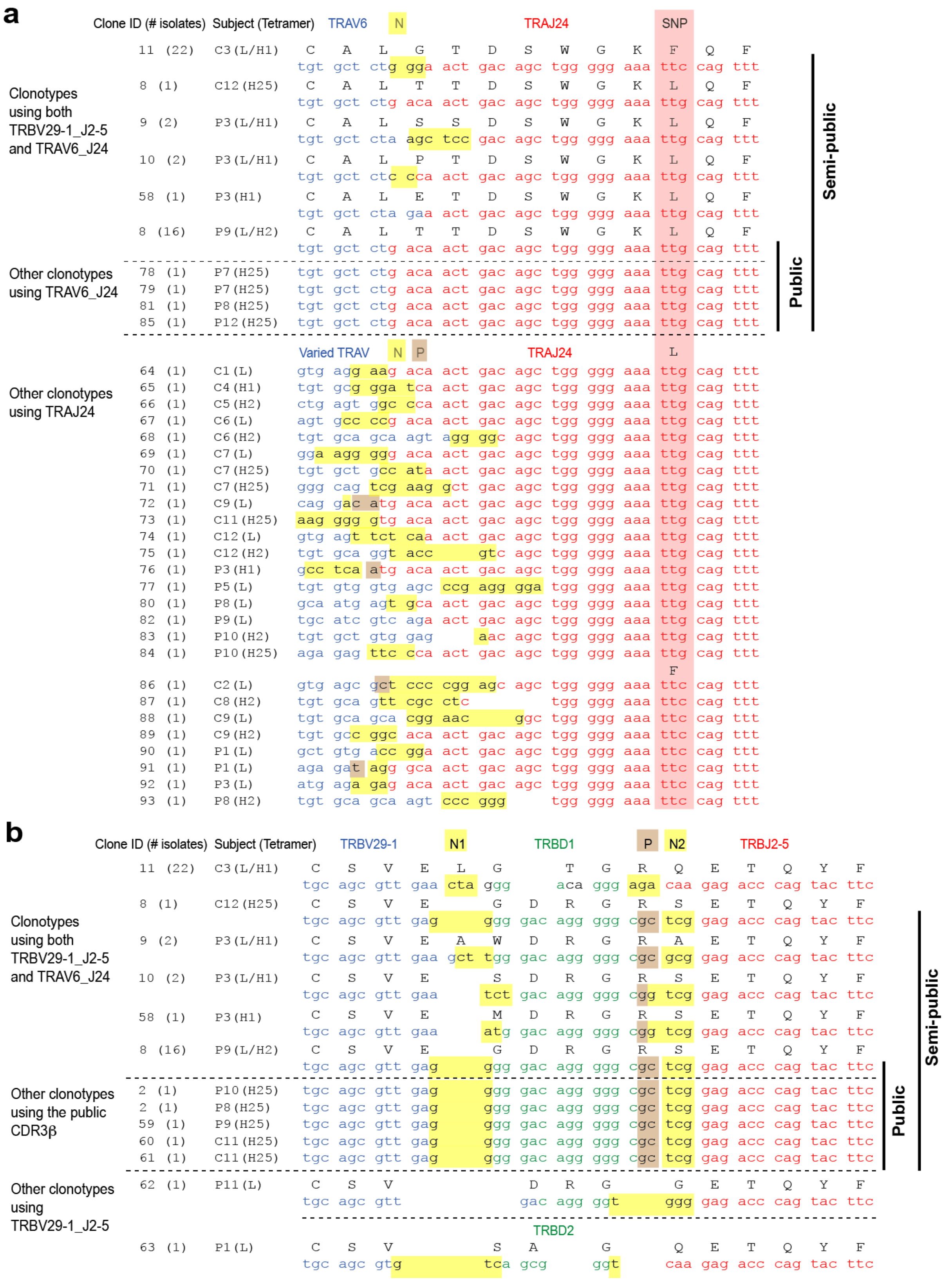

Supplementary Fig. 6 Nucleic acid sequences encoding CDR3s of clonotypes using TRAJ24 and/or TRBV29-1_J2-5 genes. Clone ID is uniquely assigned to each clonotype (see Supplementary Data 3). N (yellow) or P (brown) nucleotides are highlighted. V, D, and J gene segments are indicated in blue, green, and red, respectively. The codon containing the SNP in TRAJ24 is also highlighted in red. Clonotypes with clone ID 71, 77, 79, 81, and 86 used TRAJ24 gene in the alternative TCRα chain.

a

Tetramers (L: DQ6-HCRT1-13 H25: DQ6-HCRT25-37 H2: DQ6-HCRT87-100)

TCR ID	CloneID (#isolates)	Subject (Tetramer)	TRBV_TRBJ	CDR3β	TRAV_TRAJ	CDR3α
TCR2	89 (1)	C5 (L)	TRBV25-1_TRBJ1-1	CASESPQRGGNTEAFF	TRAV10_TRAJ18	CVVSDRGSTLGRLYF
TCR5	90 (1)	P5 (L)	TRBV25-1_TRBJ1-2	CASSAIDGEYGYTF	TRAV10_TRAJ18	CVVSDRGSTLGRLYF
TCR6	4 (2)	C12, P11 (H25)	TRBV25-1_TRBJ2-7	CASSEGQETYEQYF	TRAV10_TRAJ18	CVVSDRGSTLGRLYF
TCR7	94 (1)	P6 (H2)	TRBV25-1_TRBJ2-1	CASSEPGSYNEQFF	TRAV10_TRAJ18	CVVSDRGSTLGRLYF
TCR8	98 (1)	P6 (H2)	TRBV25-1_TRBJ1-5	CASSEYSLNQPQHF	TRAV10_TRAJ18	CVVSDRGSTLGRLYF
TCR9	5 (2)	P6 (L), P8 (H25)	TRBV25-1_TRBJ1-5	CASSEGTGGSFTQPQHF	TRAV10_TRAJ18	CVVSDRGSTLGRLYF
TCR10	100 (1)	P6 (L)	TRBV25-1_TRBJ1-6	CASSSGTGGSPLHF	TRAV10_TRAJ18	CVVSDRGSTLGRLYF
TCR11	93 (1)	P7 (H25)	TRBV25-1_TRBJ2-1	CASSEGGQVPYNEQFF	TRAV10_TRAJ18	CVVSDRGSTLGRLYF
TCR12	95 (1)	P8 (L)	TRBV25-1_TRBJ1-1	CASSESQTGSTEAFF	TRAV10_TRAJ18	CVVSDRGSTLGRLYF
TCR17	91 (1)	P8 (L)	TRBV25-1_TRBJ2-5	CASSDLRKASGRHGEETQYF	TRAV10_TRAJ18	CVVSDRGSTLGRLYF
TCR18	3 (2)	C12 (H25), P8 (L)	TRBV25-1_TRBJ2-3	CASSDPGRGTDTQYF	TRAV10_TRAJ18	CVVSDRGSTLGRLYF
TCR19	6 (4)	C7, P10 (H25), P8 (L)	TRBV25-1_TRBJ2-7	CASSEKRDNYEQYF	TRAV10_TRAJ18	CVVSDRGSTLGRLYF
TCR20	96 (1)	P8 (L)	TRBV25-1_TRBJ2-6	CASSETRVSGANVLTF	TRAV10_TRAJ18	CVVSDRGSTLGRLYF
TCR21	97 (1)	P8 (L)	TRBV25-1_TRBJ2-2	CASSEVGGGSPGELFF	TRAV10_TRAJ18	CVVSDRGSTLGRLYF
TCR22	99 (1)	P8 (L)	TRBV25-1_TRBJ2-1	CASSGISGRVPEYNEQFF	TRAV10_TRAJ18	CVVSDRGSTLGRLYF
TCR24	92 (1)	P10 (L)	TRBV25-1_TRBJ2-7	CASSEDKTYEQYF	TRAV10_TRAJ18	CVVSDRGSTLGRLYF
not tested	105 (1)	P5 (L)	TRBV25-1_TRBJ2-2	CASSAGQGGTGELFF	TRAV10_TRAJ18	CVTSDRGSTLGRLYF

b

Disease	# Subjects	# Clonotypes (all cells)	Frequency	HLA-DQB type	# Clone
Narcolepsy	8	23 (3893)	0.59	8 (DQB1*0602+)	
M. tuberculosis	7	29 (5262)	0.55	4 (DQB1*0602+)<br>1 (DQB1*0603+)<br>2 (DQB1*06-)	7<br>13<br>9

c

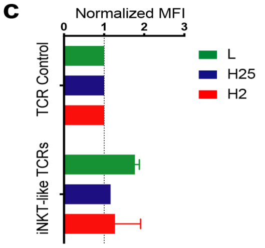

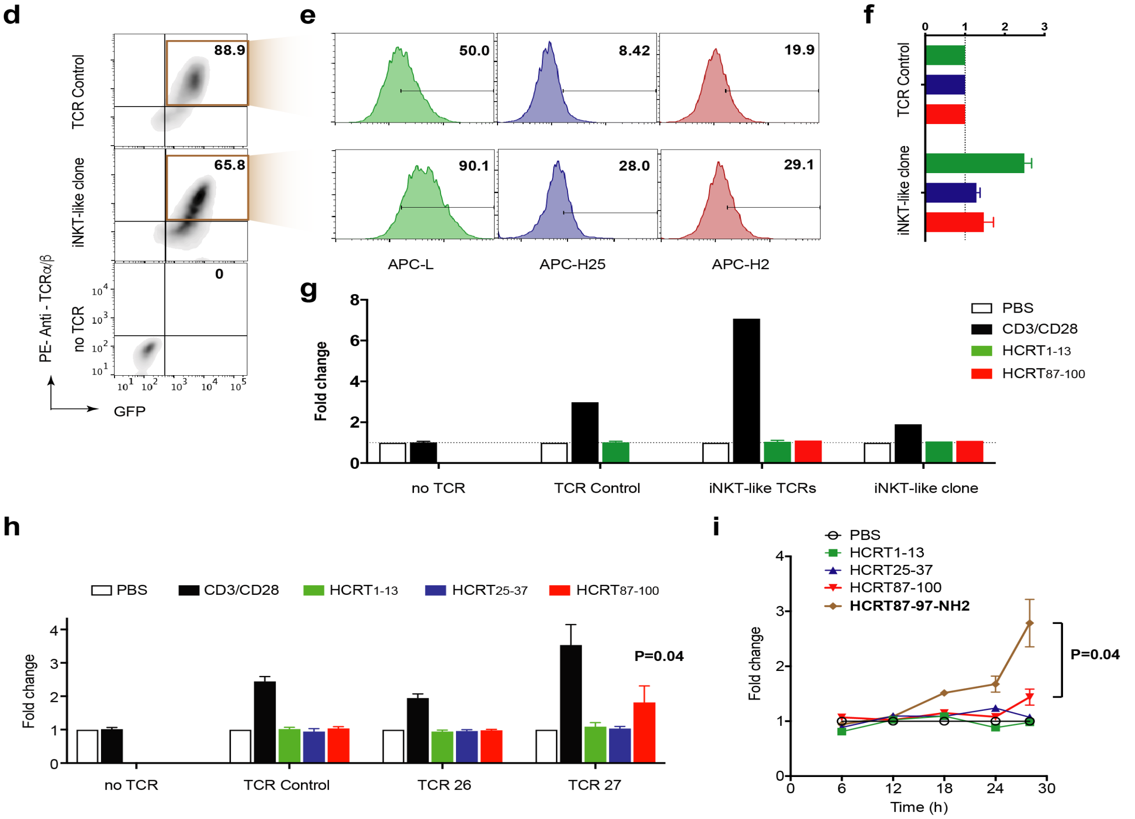

Supplementary Fig. 7 iNKT-like Clonotypes lacked clonal expansion and were unable to respond to HCRT peptides. (**a**) iNKT-like TCRs identified from DQ6[+] donors. Only TCR19 from C7 was *in vivo* expanded (2 isolates). (**b**) Comparable numbers and frequencies of iNKT-like TCRs found in two datasets obtained by single cell sorting and TCR sequencing: one described in this manuscript and one in which T cells activated by *M. tuberculosis* peptides were isolated[3]. (**c**) Nucleofection of Jurkat cells using a mixture of plasmids, each directing the expression of one TCR in (a), was used to construct a library of iNKT-like TCR transfectants. Tetramer staining of the resultant transfectant library was analyzed as in Fig. 6d. (d) A single clone expressing an iNKT-like TCR was FACS-sorted from the library and expanded. The dot-plot shows the expression of the iNKT-like TCR in this clonal line. (e and f) Tetramer staining of the clonal line was analyzed as in Fig 6c,d. (g) HCRT epitopes were unable to trigger signaling in these iNKT-like TCR trasfectants. (h) Similar experiments as shown in Fig. 6e were performed using $HCRT_{87-100}$ instead of $HCRT_{87-97}$-NH2. (i) The TCR-27 Jurkat-transfectant was tested in response of $HCRT_{87-97}$-NH2 compared to $HCRT_{87-100}$ using the luciferase-reporter assay and the responses were found to be time-dependent. Data are represented as mean ± SEM; n=3. Significance (P<0.05, bolded) is determined using *t*-test.

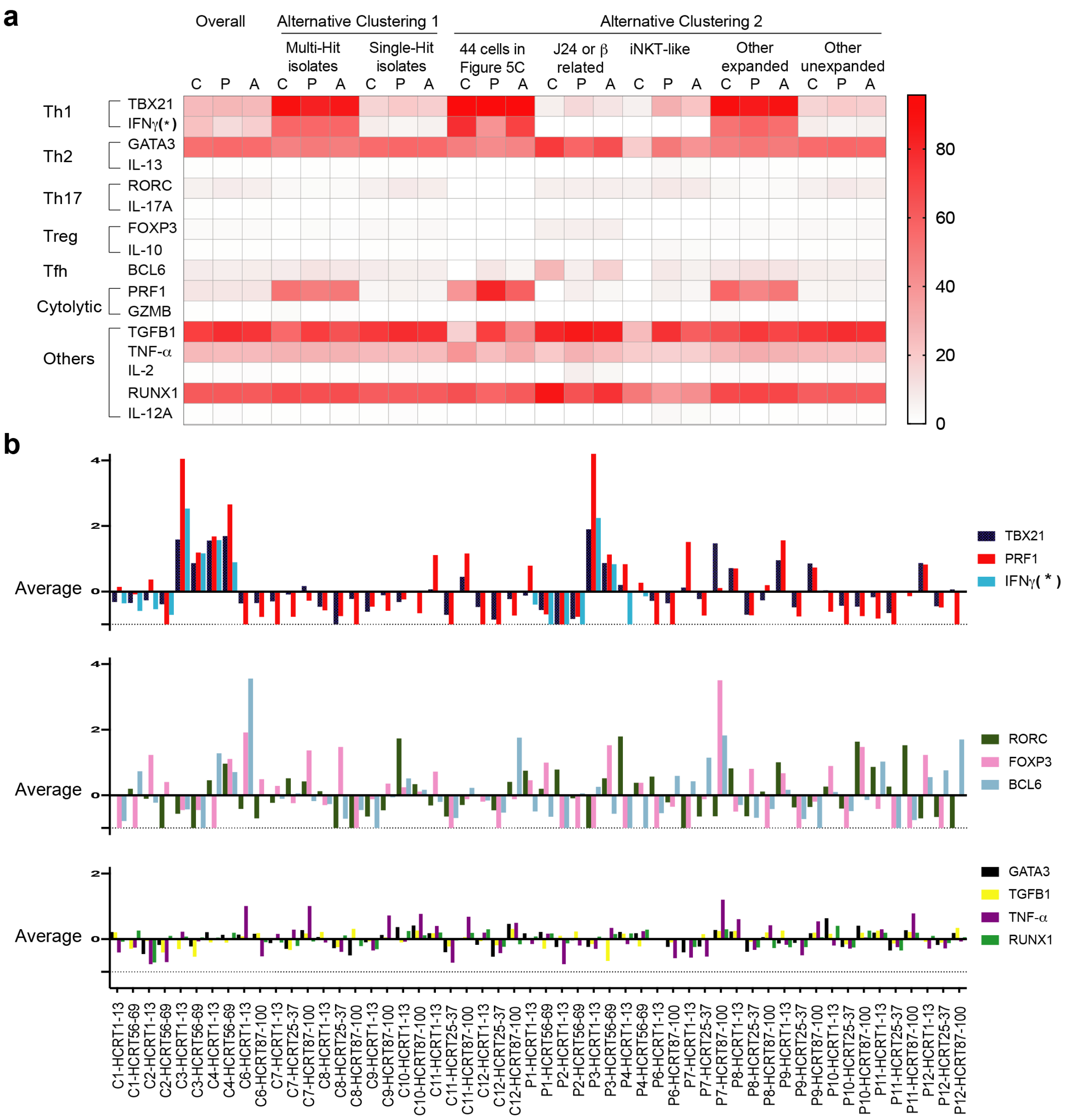

Supplementary Fig. 8 Phenotypic analysis. (**a**) Frequencies of sequenced cells from controls (C) or patients (P) or altogether (A) expressing the indicated transcript either un-clustered (the left three columns) or clustered (the result columns) as indicated (see Supplementary Data 5b). The two clustering methods shown here are different from the clustering that differentiates expanded and unexpanded clonotypes, shown in Fig. 7. Multi-hit isolates include expanded clonotypes and unexpanded but public clonotypes. (**b**) Frequencies of cells from each subject-tetramer category expressing the indicated transcript (color-coded) were compared with the average (set to zero) for the same transcript. TBET (TBX21), PRF1, and IFN-γ share very similar patterns. The detection frequencies of GATA3, TNF-α, TGF-β, and RUNX1 in all categories are around the average level (see Supplementary Data 5c).

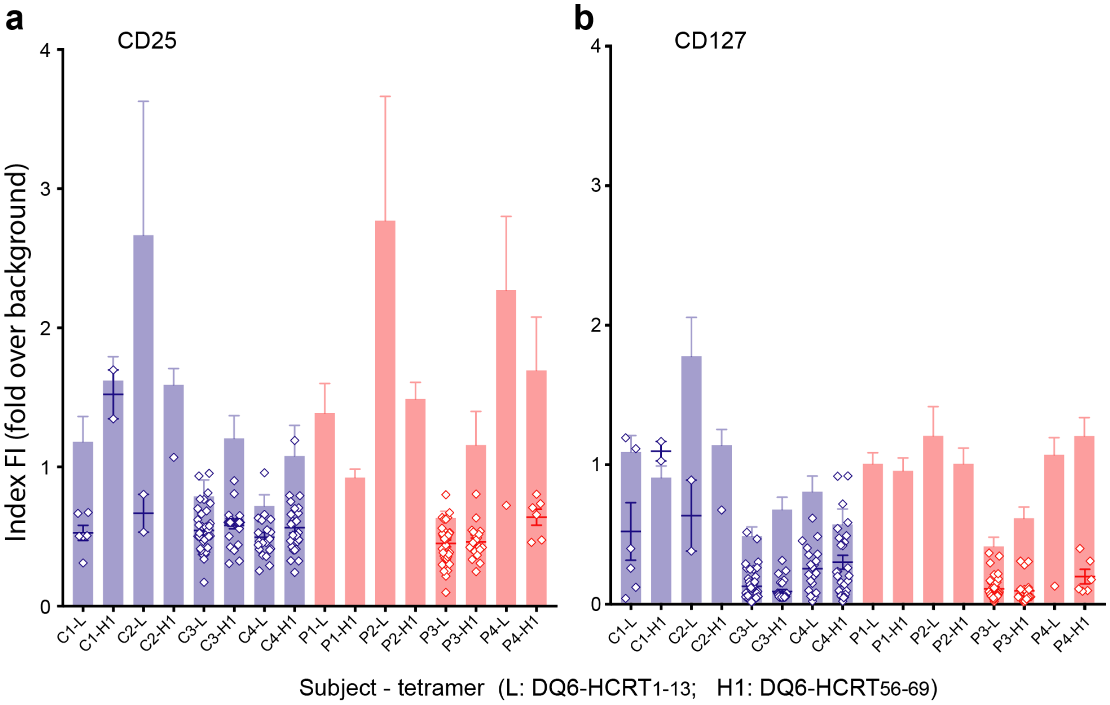

Supplementary Fig. 9 Expression of (a) CD25 and (b) CD127 on the surface of sequenced cells from Set A donors. Bar graphs represent the mean ± SEM FI of Ab staining signal on the surface of sequenced single cells in the indicated subject-tetramer category (Table 1). Each open symbol represents the index FI of Ab staining signal on the surface of one expanded cell identified from the same subject-tetramer category as the corresponding bar graph. Control: blue, patient: red. All staining is at the background level.

Supplementary Table 1 Data collection and refinement for crystallization of DQ6-HCRT$_{56\text{-}69}$.

	DQ6-HCRT$_{56\text{-}69}$
**Data collection**	
Space group	C 1 2 1
Cell dimensions	
a, *b*, *c* (Å)	63.67, 89.60, 90.93
α, β, γ (°)	90, 99.52, 90
Resolution (Å)	42.21-2.0 (2.07-2.0)*
R_{merge}	0.12 (0.95)
$CC_{1/2}$	0.99 (0.82)
$I / \sigma I$	5.68 (1.22)
Completeness (%)	99.5 (98.9)
Redundancy	3.8 (3.5)
**Refinement**	
Resolution (Å)	42.21-2.0
No. reflections	33,905 (3356)
R_{work}	22.2 (38.3)
R_{free}	25.6 (39.1)
No. atoms	
Protein	3036
Ligand/ion	64
Water	272
B-factors	
Protein	39.2
Peptide	35.7
Ligand/ion	82.5
Water	42.0
R.m.s. deviations	
Bond lengths (Å)	0.014
Bond angles (°)	1.57

*Values in parentheses are for highest-resolution shell.

Supplementary References:

1 Jensen, K. K. *et al.* Improved methods for predicting peptide binding affinity to MHC class II molecules. *Immunology* **154**, 394-406, doi:10.1111/imm.12889 (2018).

2 Siebold, C. *et al.* Crystal structure of HLA-DQ0602 that protects against type 1 diabetes and confers strong susceptibility to narcolepsy. *Proc Natl Acad Sci U S A* **101**, 1999-2004, doi:10.1073/pnas.0308458100 (2004).

3 Glanville, J. *et al.* Identifying specificity groups in the T cell receptor repertoire. *Nature* **547**, 94-98, doi:10.1038/nature22976 (2017).
