# Supplementary material for: In vivo clonal expansion and phenotypes of hypocretin-specific CD4+ T cells in narcolepsy patients and controls
Source: Nat Commun. 2019 Nov 20;10:5247. doi: 10.1038/s41467-019-13234-x (PMC6868281; doi:10.1038/s41467-019-13234-x)
Supplement: Supplementary file 9 — Reporting Summary [file 41467_2019_13234_MOESM9_ESM.pdf]

nature research

Corresponding author(s): Wei Jiang & Elizabeth D. Mellins

Last updated by author(s): Oct 17, 2019

Reporting Summary

Nature Research wishes to improve the reproducibility of the work that we publish. This form provides structure for consistency and transparency in reporting. For further information on Nature Research policies, see Authors & Referees and the Editorial Policy Checklist.

Statistics

For all statistical analyses, confirm that the following items are present in the figure legend, table legend, main text, or Methods section.

n/a	Confirmed	
☐	☒	The exact sample size (n) for each experimental group/condition, given as a discrete number and unit of measurement
☐	☒	A statement on whether measurements were taken from distinct samples or whether the same sample was measured repeatedly
☐	☒	The statistical test(s) used AND whether they are one- or two-sided<br>*Only common tests should be described solely by name; describe more complex techniques in the Methods section.*
☐	☒	A description of all covariates tested
☐	☒	A description of any assumptions or corrections, such as tests of normality and adjustment for multiple comparisons
☐	☒	A full description of the statistical parameters including central tendency (e.g. means) or other basic estimates (e.g. regression coefficient) AND variation (e.g. standard deviation) or associated estimates of uncertainty (e.g. confidence intervals)
☐	☒	For null hypothesis testing, the test statistic (e.g. F, t, r) with confidence intervals, effect sizes, degrees of freedom and P value noted<br>*Give P values as exact values whenever suitable.*
☒	☐	For Bayesian analysis, information on the choice of priors and Markov chain Monte Carlo settings
☒	☐	For hierarchical and complex designs, identification of the appropriate level for tests and full reporting of outcomes
☒	☐	Estimates of effect sizes (e.g. Cohen's d, Pearson's r), indicating how they were calculated

Our web collection on statistics for biologists contains articles on many of the points above.

Software and code

Policy information about availability of computer code

Data collection	X-ray diffraction data were recorded at the LRL-CAT 31-ID beamline Advanced Photon Source (APS) in Chicago. Index single cell sorting data were collected using FACSDiva at the Stanford FACS facillity. High throughput sequencing was performed using Illumina Miseq at the Stanford Human Immune Monitoring Center.
Data analysis	Software and code used in this study are all described in method. These include an open source server NetMHCIIpan 3.2 for HLA class II peptide binding analysis, softwares used in crystallization and structure determination, FlowJo used for flow cytometric analysis, GraphPad Prism used for statistical analysis, and previously published custom codes for single cell sequencing analysis (see below, #1 and #2).<br>#1: Han, A., Glanville, J., Hansmann, L. & Davis, M. M. Linking T-cell receptor sequence to functional phenotype at the single-cell level. Nat Biotechnol 32, 684-692, doi:10.1038/nbt.2938 (2014).<br>#2: Glanville, J. et al. Identifying specificity groups in the T cell receptor repertoire. Nature 547, 94-98, doi:10.1038/nature22976 (2017).

For manuscripts utilizing custom algorithms or software that are central to the research but not yet described in published literature, software must be made available to editors/reviewers. We strongly encourage code deposition in a community repository (e.g. GitHub). See the Nature Research guidelines for submitting code & software for further information.

Data

Policy information about availability of data

All manuscripts must include a data availability statement. This statement should provide the following information, where applicable:

- Accession codes, unique identifiers, or web links for publicly available datasets
- A list of figures that have associated raw data
- A description of any restrictions on data availability

X-ray structural data for DQ6-HCRT56-69 crystallization has been deposited to worldwide protein data bank (https://www.rcsb.org), PDBID: 6GIG; and the structure has been validated. Raw single-cell sequencing data has been deposited to NCBI GEO database (GSE135852). Processed sequencing data are provided in

Supplementary Data 2. All relevant data are available from the authors.

Field-specific reporting

Please select the one below that is the best fit for your research. If you are not sure, read the appropriate sections before making your selection.

☒ Life sciences ☐ Behavioural & social sciences ☐ Ecological, evolutionary & environmental sciences

For a reference copy of the document with all sections, see nature.com/documents/nr-reporting-summary-flat.pdf

Life sciences study design

All studies must disclose on these points even when the disclosure is negative.

Sample size	For the single cell analysis, 12 narcoleptic patients and 12 allele-matched controls were randomly paired. Set A of 4 pairs were stained with the DQ6 tetramer in complex with one of the following peptides: HCRT1-13, and HCRT56-69; Set B of 8 pairs were stained with the DQ6 tetramer in complex with one of the following peptides: HCRT1-13, HCRT25-37, and HCRT87-100. This represents a good sampling pool for an exploratory study of narcolepsy, considering both the prevalence of this disease and sample sizes used in previous reports. We also considered the practical number of purified CD4+ T cells (20-30 millions) in each available donor PBMC sample (50-100 millions) and the necessary starting cell number (5-10 millions) that allows the staining and sorting of one 96-well plate of tetramer positive single cells among purified CD4+ T cells (1-100 in a million cells) for single cell analysis at each tetramer specificity. This leads to the design that each donor sample will be used in 2-3 staining experiments each with one of the above tetramers.
Data exclusions	The frequency of expanded DQ6-HCRTHCRT87-100 tetramer+ cells in subjects who had received a recent TIV vaccination prior to blood draw (C5, C6, C11, P5) was significantly higher than in controls who had received no influenza vaccination (C9, C10) or received H1N1 vaccination ~5 years prior to blood draw during the 2009 flu pandemic (C7, C8, C12), P=0.0476 in the Mann-Whitney U test or P<0.0001 in a chi-squared test. Therefore, we showed the comparison of case/control cells with DQ6-HCRT87-100 tetramer specificity either using all subjects or excluding TIV-vaccinated subjects to minimize biases caused by recent irrelevant triggering of in vivo expansion of cross-binding T cell clones.
Replication	All experiments have been performed with appropriate replicates as described in figures and methods. Replications of key results are all confirmed.
Randomization	Donor PBMC samples were paired into 12 groups; each group contains a patient sample and a control sample. The pairing of samples is random.
Blinding	Yes. All authors who performed the single cell sequencing analysis are blinded to donor samples that have been randomized into patient/ control pairs per sorting experiment.

Reporting for specific materials, systems and methods

We require information from authors about some types of materials, experimental systems and methods used in many studies. Here, indicate whether each material, system or method listed is relevant to your study. If you are not sure if a list item applies to your research, read the appropriate section before selecting a response.

Materials & experimental systems

n/a	Involved in the study	
☐	☒	Antibodies
☐	☒	Eukaryotic cell lines
☒	☐	Palaeontology
☒	☐	Animals and other organisms
☐	☒	Human research participants
☒	☐	Clinical data

Methods

n/a	Involved in the study	
☒	☐	ChIP-seq
☐	☒	Flow cytometry
☒	☐	MRI-based neuroimaging

Antibodies

Antibodies used	The anti-DQ antibody SPV-L3 was purified by the Mellins Laboratory. Alexa fluor 488 anti-human CD4 (Cat# 300519), PerCP-Cy5.5 anti-human CD19 (Cat# 302230), APC anti-human CD127 (Cat# 351316), and APC/CY7 anti-human CD25 (Cat# 302614) antibodies were purchased from BioLegend. PE anti-TCRα/β (Cat# 555548) was purchased from BD Biosciences.
Validation	The activity of SPV-L3 anti-DQ antibody in the ascites format or purified from mouse hybridoma was confirmed by ELISA. Commercial available antibodies were provided by vendors with validated function and specificity.

Eukaryotic cell lines

Policy information about cell lines

Cell line source(s)	The APC cell line T2DQ6 was previously constructed by the Mellins Laboratory; The Jurkat line J76-NFATRe-Luc and the APC line K562-DQ6 were provided by the Davis Laboratory. All J76-NFATRe-Luc cell transfectants mentioned are constructed in this study.
Authentication	N/A
Mycoplasma contamination	All cell lines were tested (by PCR) negative or treated with plasmocin to completely eliminate Mycoplasma contamination if any prior to culturing and cell activation assays.
Commonly misidentified lines (See ICLAC register)	N/A

Human research participants

Policy information about studies involving human research participants

Population characteristics	All donors in this study are DQ6-allele matched. Narcoleptic patients with cataplexy met the criteria for International Classification of Sleep Disorders 3 (ICSD3) for type 1 narcolepsy. Some individuals have received influenza vaccination previously, and the time interval from vaccination to blood draw have been reported.
Recruitment	PBMCs were received from the Stanford Center for Sleep Sciences and Medicine without potential self-selection bias.
Ethics oversight	Written consent was obtained in all cases under a Stanford Institutional Review Board approved protocol, following the guidelines for human subjects' research under U.S. Department of Health and Human Services human subjects regulations (45 CFR Part 46).

Note that full information on the approval of the study protocol must also be provided in the manuscript.

Flow Cytometry

Plots

Confirm that:

☒ The axis labels state the marker and fluorochrome used (e.g. CD4-FITC).

☒ The axis scales are clearly visible. Include numbers along axes only for bottom left plot of group (a 'group' is an analysis of identical markers).

☒ All plots are contour plots with outliers or pseudocolor plots.

☒ A numerical value for number of cells or percentage (with statistics) is provided.

Methodology

Sample preparation	CD4+ T cells were isolated from donor PBMCs using magnetic-activated cell sorting and labeled with antibodies and tetramers before its analysis on a flow cytometer or a sorter.
Instrument	FACSAriaII was used for single cell sorting; FACSCalibur and LSRII were used for flow cytometric analysis. The Vendor for all cytometers is BD Biosciences.
Software	FACSDiva (BD Biosciences) was used to acquire the flow cytometric data. FlowJo (Tree Star Software) was used to analyze the data.
Cell population abundance	The frequency of tetramer positive CD4+ T cells among all circulating CD4+ T cells is at the range of 1-100 per million. This is suggested by previous reports and validated in our study. Sorting of the tetramer positive population was based on stringent gating with 99% accuracy.
Gating strategy	FSC/SSC gating, singlet gating, dead cell exclusion were performed for each sample. Details in Supplementary Figure 4.

☒ Tick this box to confirm that a figure exemplifying the gating strategy is provided in the Supplementary Information.
